# Synthesis and Properties Study of Liquid Crystalline Epoxy Resin Containing Azomethine-Based Monomeric Mesogens

**DOI:** 10.3390/polym17192632

**Published:** 2025-09-29

**Authors:** Junjie Huang, Hongmei Pan, Deliang Le, Zengxin Ouyang, Kemei Pei

**Affiliations:** College of Chemistry and Chemical Engineering, Zhejiang Sci-Tech University, Hangzhou 310018, China; 202130107300@mails.zstu.edu.cn (J.H.); pan150_8280@163.com (H.P.); 2023221002026@mails.zstu.edu.cn (D.L.); 2023211001033@mails.zstu.edu.cn (Z.O.)

**Keywords:** liquid crystal epoxy resin, crystallinity, fracture toughness, thermal conductivity

## Abstract

The epoxy monomer N,1-bis(4-(2-oxiranemethoxy)phenyl)methylamine (HBAP-EP) was synthesized through the Schiff base reaction and epichlorohydrin method, and the HBAP-EP monomer was cured using p-aminobenzene sulfonamide (SAA). Differential scanning calorimetry (DSC), X-ray diffraction (XRD), and polarizing optical microscopy (POM) demonstrated that the epoxy monomer exhibits reversible liquid crystal properties, and the liquid crystal fraction of the monomer can reach 14.4% after curing at 120 °C. The fracture toughness of the resin cured at 120 °C can reach 0.93 KJ·m^−2^, and its thermal conductivity is 0.3229 W·(m·K)^−1^, both of which are higher than those of ordinary epoxy resin.

## 1. Introduction

Epoxy resin dominates the majority of the thermosetting resin market, owing to its exceptional bonding, mechanical, and thermal properties, coupled with its superior chemical stability, low shrinkage rate, and electrical insulation [1,2]. It is frequently utilized in various industrial fields, including adhesives, coatings, electronic packaging materials, and composite materials. In the realm of electronic device production, particularly in technological innovations involving precise electronic components, electronic components are evolving towards higher integration, miniaturization, and multifunctionality. This inevitably leads to heat accumulation within the materials during device operation, compromising the stability of the electronic components and shortening their lifespan [3,4,5]. The traditional epoxy resin exhibits an intrinsic thermal conductivity of merely 0.2 W·m^−1^·K^−1^, which no longer satisfies the requirements of modern advanced materials [6,7].

Many researchers enhance thermal conductivity by incorporating fillers, yet fillers inevitably have a negative impact on the overall mechanical strength, flexibility, and transparency of the material [8,9,10]. Meanwhile, researchers have demonstrated that the thermal conductivity of polymer composites is primarily influenced by the polymer matrix, rather than the thermal conductive fillers [11]. Therefore, by improving the microstructure within the resin to suppress phonon scattering and enhance its intrinsic thermal conductivity, it is a more feasible solution to achieve breakthroughs in composite material performance [12,13]. They introduced mesogenic units into the structure of epoxy monomers, leading to the formation of local crystalline structures inside upon curing. This ordered microstructure reduces molecular defects, enhancing the coordination of vibrations between molecules and between lattices. Consequently, phonon scattering is suppressed, the number of phonon non-mean-free paths increases, and ultimately, the intrinsic thermal conductivity of the epoxy resin is improved [14,15].

The introduction of imine bonds modulates the length and rigidity of the entire linker fragment, thereby regulating liquid crystal behavior and physical properties [16]. Takezawa et al. began with the molecular structure design of liquid crystal epoxy monomers, utilizing diphenyl benzoate as an intermediate. By altering the length of the flexible segment on the main chain, they synthesized various epoxy monomers. The intrinsic thermal conductivity of the cured epoxy monomers was approximately five times higher than that of ordinary epoxy resins [17]. Anber et al. synthesized a novel liquid crystalline diol by reacting bisphenol A diglycidyl ether (DGEBA) with a new type of liquid crystalline diol. The study revealed that, compared to conventional amine systems, the cured polymer exhibited high flexibility and excellent adhesive properties [18]. To establish an ordered microstructure and suppress phonon scattering, Hossain and his team optimized and synthesized a liquid crystalline epoxy resin monomer capable of topological chemical polymerization, featuring diphenyl diacetylene as the mesogenic unit. This monomer is capable of forming a microscopically ordered structure and an intermolecular π-conjugated network, both of which can synergistically suppress phonon scattering [19]. The synthesis of imine bonds is simple, the sources of raw materials are extensive, and they possess excellent physicochemical properties, making them favored by many researchers [20,21]. Kim et al. designed and synthesized three liquid crystalline epoxy monomers based on azomethine mesogenic units and studied the thermal conductivity of the resins cured from these three monomers. The results indicated that the thermal conductivity of liquid crystalline epoxy resin is closely correlated with its main chain structure. The higher the orderliness of the main chain structure, the higher the intrinsic λ of the liquid crystalline epoxy resin [22,23].

The primary driving forces behind liquid crystalline epoxy resins are the π-π stacking interactions and the regulatory effect of imine bonds on liquid crystal properties [24,25]. The Schiff base reaction involves the reaction of aldehydes with primary amines to form compounds containing imine bonds (−C=N−), which exhibit numerous excellent properties [26]. To this end, an epoxy monomer containing imine bonds was synthesized via the Schiff base reaction and epichlorohydrin method. p-Aminobenzene sulfonamide (SAA) served as the curing agent to cure the epoxy monomer. The cured resin demonstrates significantly enhanced thermal conductivity compared to ordinary epoxy resin and possesses excellent mechanical properties.

## 2. Materials and Methods

### 2.1. Materials

p-Hydroxybenzaldehyde, p-aminophenol, p-toluenesulfonic acid, anhydrous ethanol, epichlorohydrin (ECH), tetrabutylammonium bromide (TBAB), sodium hydroxide (NaOH), p-aminobenzenesulfonamide (SAA), 1,4-dioxane, potassium hydrogen phthalate, cresol red, and phenolphthalein were all sourced from Shanghai Maclin Biochemical Technology Co., Ltd. in Shanghai, China. Concentrated hydrochloric acid was obtained from Huzhou Shuanglin Chemical Technology Co., Ltd. in Huzhou, China. Deionized water was prepared in our laboratory.

### 2.2. Synthesis and Curing of Epoxy Monomer HBAP-EP

#### 2.2.1. Synthesis of 4-(4-Hydroxybenzylideneamino)phenol (HBAP)

**Figure 1:**
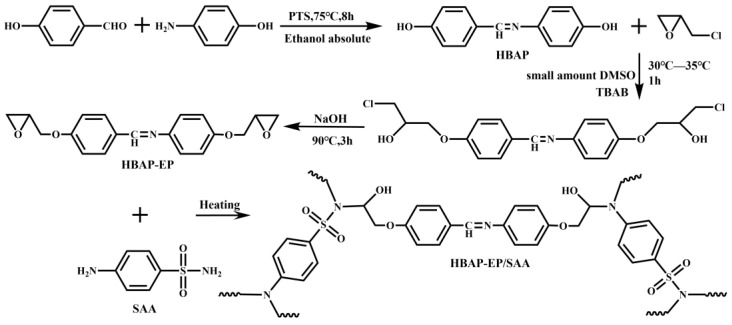
Reaction synthesis route and curing process of HBAP-EP.

The reaction synthesis route and curing scheme are illustrated in Figure 1. First, 0.1 mol of p-hydroxybenzaldehyde (12.21 g), 0.1 mol of p-aminophenol (10.91 g), and 0.6936 g of p-toluenesulfonic acid (3% of the total reactant mass) were sequentially added to a four-neck flask equipped with a stirrer, condenser tube, and thermometer. A 100 mL quantity of anhydrous ethanol was then added to the flask to dissolve the reactants, which were subsequently placed in an oil bath maintained at 75 °C. After stirring at a constant temperature for 8 h, the reactants were poured into deionized water, and a yellow solid was obtained via suction filtration. This solid was then placed in a 60 °C forced-air oven and dried for 24 h to yield 20.37 g of compound HBAP, with a yield of 95.61%.

#### 2.2.2. Synthesis of N,1-Bis(4-(2-methoxyethoxy)phenyl)methylamine (HBAP-EP)

The epoxy monomer HBAP-EP was synthesized via the epichlorohydrin method. To a 500 mL four-neck flask equipped with a thermometer, stirrer, and condenser tube, 0.1 mol of compound HBAP (21.30 g), 4.0 mol of ECH (374.08 g), and 0.01 mol of TBAB (3.224 g) were added. Then, a small amount of DMSO was added to dissolve the solid completely. After reacting at 30–35 °C for 1 h, the temperature was raised to 90 °C. A 50% aqueous solution of sodium hydroxide was added, and the reaction was allowed to proceed for 3 h before it ended. Most of the ECH was removed via rotary evaporation, and the residue was washed with an ethanol aqueous solution. After filtration with suction, 29.66 g of white solid was obtained, with a yield of 91.24%.

#### 2.2.3. Curing of Epoxy Monomer HBAP-EP

HBAP-EP and SAA were weighed in a stoichiometric ratio corresponding to equal amounts of active hydrogen and epoxy groups. They were mixed uniformly in an agate mortar, and the mixture was then transferred into a polytetrafluoroethylene mold. The mold was placed in a forced-air oven at 120 °C, 140 °C, and 160 °C, respectively, and heated for curing for 2 h. The cured epoxy resin is hereinafter referred to as HBAP-EP/SAA.

### 2.3. Characterization

Nuclear magnetic resonance spectroscopy (NMR) was conducted on the sample using a Bruker Advance 400 NMR spectrometer from Bruker Corporation in Ettlingen, Germany, with CDCl_3_ or DMSO-D_6_ as the solvent and TMS as the internal reference.

The infrared spectrum was measured using a Nicolet IS10 Fourier Transform Infrared Spectrometer (FT-IR) from Nicolet, in Madison, WI, USA. The solid sample was ground into a fine and uniform powder using a mortar and then tested in ATR mode. The wavenumber acquisition range spanned from 400 to 4000 cm^−1^, with 32 scans and a resolution of 4 cm^−1^.

Thermal analysis was conducted using a Q2000 differential scanning calorimeter (DSC) from TA Instruments, located in Newcastle, DE, USA, to determine the phase transition temperature. The test was performed by heating the sample from 50 °C to 250 °C at a rate of 5 °C/min in a nitrogen atmosphere, with a flow rate maintained at 60 mL/min.

The polarization microscope (POM) test utilizes a model LV100 polarization microscope from NIKON Corporation in Tokyo, Japan, equipped with a Linkam PE100 industry-specific polarization hot stage and 5×, 10×, 20×, and 50× ultra-long working distance polarization objectives. After thoroughly grinding the HBAP-EP powder in a mortar, it was placed between a glass slide and a cover slip. Then, it was placed on a hot stage to observe the changes in optical properties during the heating and cooling processes. The heating and cooling rates were maintained at 2 °C·min^−1^, with a temperature range of 25~180 °C. An appropriate amount of HBAP-EP/SAA mixed powder in stoichiometric ratio was placed on a glass slide, covered with a cover glass, and then placed in an oven for curing. After the sample was fully cured, the crystal texture of the cured resin was observed using a Polarizing Optical Microscope (POM).

The X-ray diffraction pattern was obtained using an X-ray diffractometer (XRD) of model D8Quest from Bruker AXS GmbH, Ettlingen, Germany. The diffraction pattern was obtained by irradiating with Cu-Ka (wavelength λ = 0.15406 nm) radiation, filtered through a graphite filter, under the conditions of an instrument operating at a voltage of 40 KV and a current of 30 mA. The thin-film or powder sample was placed on a quartz XRD stage and scanned from 5° to 50° at a scanning speed of 2° · min^−1^.

The lap tensile shear strength was tested using an EZ Test series universal material testing machine from Shimadzu Corporation in Kyoto, Japan, and the fracture toughness was measured at room temperature by employing the three-point bending method in accordance with GB4161-1984 [27].

The thermal conductivity test was conducted using a thermal constant analyzer, model TPS2500S, from the Swedish company Hot Disk (Gothenburg, Swedish). Based on the standard GB/T 32064-2015, the transient plane source method was employed to measure the thermal conductivity, specific heat capacity, and thermal diffusivity of the samples [28].

The microstructure of the tensile fracture surface of the cured resin was observed using a scanning electron microscope (SEM) of model HITACHI S 4700, sourced from Hitachi High-Technologies Corporation in Tokyo, Japan.

## 3. Results

### 3.1. Structural Characterization of HBAP-EP

**Figure 2:**
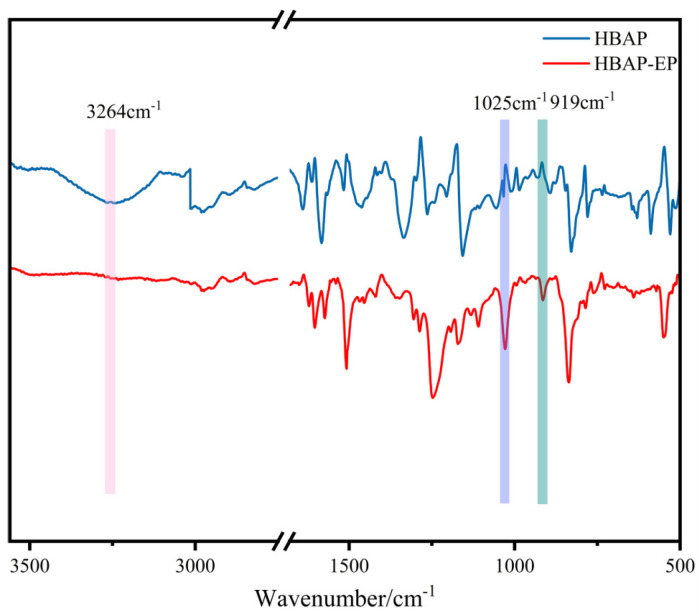
Infrared spectra of HBAP and HBAP-EP.

According to the infrared spectrum depicted in Figure 2, the presence of the characteristic peaks for the epoxy group (919 cm^−1^) and ether bond (1025 cm^−1^), coupled with the absence of the characteristic peak for the phenolic hydroxyl group (3264 cm^−1^), can preliminarily indicate the successful synthesis of the epoxy monomer [29,30].

**Figure 3:**
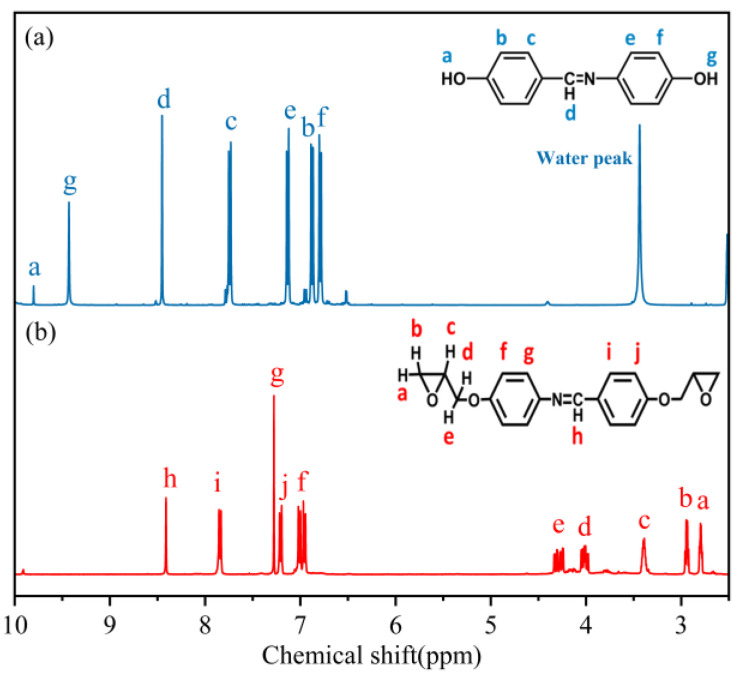
(**a**) NMR hydrogen spectrum of HBAP. (**b**) NMR hydrogen spectrum of HBAP-EP.

HBAP utilizes DMSOD_6_ as a solvent, and its chemical shift is observed at 3.30 ppm, corresponding to the water peak. As illustrated in Figure 3a, the characteristic peaks of active hydrogen on the two phenolic hydroxyl groups in HBAP are located at a (δ = 10.13 ppm) and g (δ = 9.42 ppm). The characteristic peak of hydrogen atom on the methylenimine group is observed at d (δ = 8.44 ppm). The characteristic peaks of hydrogen atoms on the benzene ring are found at b, c, e, and f (δ = 6.80–8.00 ppm).

HBAP-EP utilizes CDCl_3_ as the solvent. As depicted in Figure 3b, the characteristic peaks of hydrogen atoms on the epoxy group are observed at a, b, and c (δ = 2.81 ppm, 2.95 ppm, 3.40 ppm); the characteristic peaks of hydrogen atoms on the methylene group of the glycidyl group are located at d and e (δ = 4.01 ppm, 4.29 ppm); and the characteristic peaks of hydrogen atoms on the benzene ring are found at f, g, i, and j (δ = 6.80–8.00 ppm). In summary, the disappearance of the characteristic peak of phenolic hydroxyl groups and the emergence of the characteristic peak of epoxy groups indicate the successful preparation of the expected product, HBAP-EP.

### 3.2. HBAP-EP Epoxy Equivalent

First, prepare a 0.1% cresol red indicator solution, a 0.1% phenolphthalein indicator solution, a saturated sodium hydroxide solution, and a 0.2 mol·L^−1^ hydrochloric acid-1,4 dioxane solution for future use.

Pipette 5.4 mL of supernatant from the saturated sodium hydroxide solution, transfer it to 200 mL of deionized water, and dilute the mixture to 1000 mL with ethanol. Bake potassium hydrogen phthalate to constant weight, precisely weigh 0.7500 g, and dissolve it in 50 mL of deionized water. Add 2 drops of phenolphthalein indicator solution, titrate with the prepared sodium hydroxide–ethanol solution until the solution turns pink and maintain this color for 30 s. Simultaneously, conduct a blank experiment. Calculate the concentration of the sodium hydroxide–ethanol standard titration solution using Formula (1), with the unit being mol·L^−1^.

(1) $$C_{\left(NaOH\right)}=\frac{1000m}{(V_{1}-V_{2})M}$$

In the formula, $C_{\left(NaOH\right)}$ represents the concentration of the standard titration solution of sodium hydroxide in units of mol·L^−1^. m denotes the mass of potassium hydrogen phthalate in units of g. V_1_ signifies the volume of sodium hydroxide solution in units of mL. V_2_ indicates the volume of sodium hydroxide solution consumed in the blank test in units of mL. M stands for the molar mass of potassium hydrogen phthalate in units of g·mol^−1^.

Weigh an appropriate amount of sample into a 250 mL iodine flask; then, add 15 mL of 1,4-dioxane to the iodine flask. Dissolve the sample using ultrasonication and cool it to room temperature. Add 25 mL of hydrochloric acid-1,4-dioxane solution to a cooled iodometric flask and let it react at room temperature for 30 min. Wash the stopper and the inner wall of the iodine flask with ethanol, add three to five drops of cresol red indicator solution, and titrate with sodium hydroxide–ethanol standard titration solution. The end point of titration is indicated when the solution color changes from yellow to purple. Meanwhile, conduct a blank test. The epoxy-equivalent weight (EEW) is calculated using Formula (2).

(2) $$EEW=\frac{1000m}{(V_{0}-V)C_{\left(NaOH\right)}}$$

In the formula, EEW represents the epoxy-equivalent weight of the sample, with the unit of g·eq^−1^. m denotes the sample mass in units of g. V_0_ signifies the volume of sodium hydroxide–ethanol standard titration solution consumed in the blank test in units of mL. V stands for the volume of sodium hydroxide–ethanol standard titration solution consumed in the titration process in units of mL. $C_{\left(NaOH\right)}$ indicates the concentration of the sodium hydroxide–ethanol standard titration solution in units of mol·L^−1^.

Table 1 presents the calculated concentrations of the sodium hydroxide–ethanol standard solution. Table 2 presents three sets of parallel experiments conducted to determine the epoxy-equivalent weight of HBAP-EP. Based on the average values of the three sets of experiments, the epoxy-equivalent weight of HBAP-EP was determined to be 163.95 g·eq^−1^. The relative deviation of the range of the detection results from the average value in the three sets of parallel experiments was less than 0.8%, indicating that the results are within the allowable error range.

### 3.3. Characterization of HBAP-EP Liquid Crystal Properties

**Figure 4:**
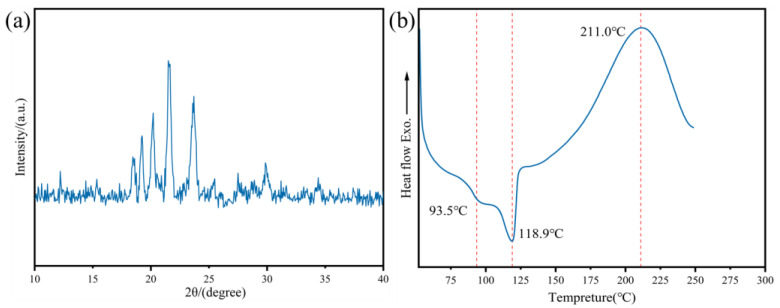
(**a**) XRD curve of HBAP-EP and (**b**) DSC curve of HBAP-EP at increasing temperature.

As shown in Figure 4a, the XRD curve of HBAP-EP exhibits sharp diffraction peaks, indicating that HBAP-EP is a standard crystalline polymer. The crystallinity of HBAP-EP, as calculated using Jade software (Jade 9.0), is 84.62%.

Observe the phase transition temperature of HBAP-EP through DSC. As shown in Figure 4b, the first endothermic peak represents the phase transition from crystalline to liquid crystal structure, while the second endothermic peak indicates the clearing point temperature, representing the second transition from anisotropic liquid crystal to isotropic liquid crystal phase [29]. The results indicate that the liquid crystal temperature range of the liquid crystal monomer HBAP-EP during the heating process is 93.5–118.9 °C. The exothermic peak at 211.0 °C in the DSC curve of HBAP-EP originates from the heat released during the addition reaction between the methylimino group and the epoxy group in HBAP-EP.

**Figure 5:**
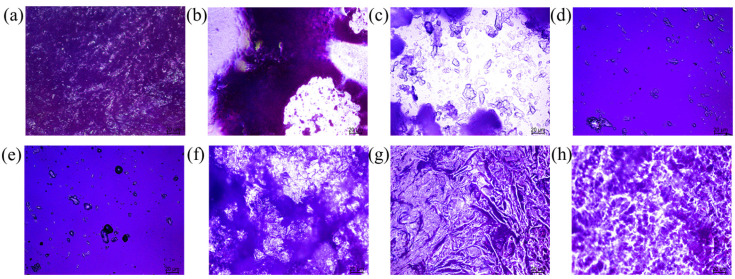
POM images of HBAP-EP during heating at (**a**) 25 °C, (**b**) 90 °C, (**c**) 120 °C, and (**d**) 180 °C, as well as cooling at (**e**) 160 °C, (**f**) 100 °C, (**g**) 90 °C, and (**h**) 25 °C.

To observe the phase transition behavior more intuitively, Figure 5 displays the POM images of HBAP-EP monomer during the heating process. At 95 °C, the nematic liquid crystal phase with a brightened visual area was observed (Figure 5b), and some crystalline parts remain at 120 °C (Figure 5c). As the temperature continues to rise, the liquid crystal phase disappears. This is due to the anionic step-growth polymerization reaction between the azomethine group and the epoxy in HBAP-EP, which causes the monomers to self-polymerize into amorphous polymers. This disrupts the π-π stacking interaction of the mesogenic groups, leading to a reduction in liquid crystal domains (Figure 5d,e). During the cooling process, the originally disappeared liquid crystal phase texture reappeared at 100 °C (Figure 5g), indicating that the HBAP-EP monomer exhibits reversible liquid crystallinity. The phase transition temperature observed through POM images is higher than that measured by DSC, which may be attributed to instrumental hysteresis.

### 3.4. HBAP-EP Thermal Curing Behavior and Cured Material Performance Characterization

**Figure 6:**
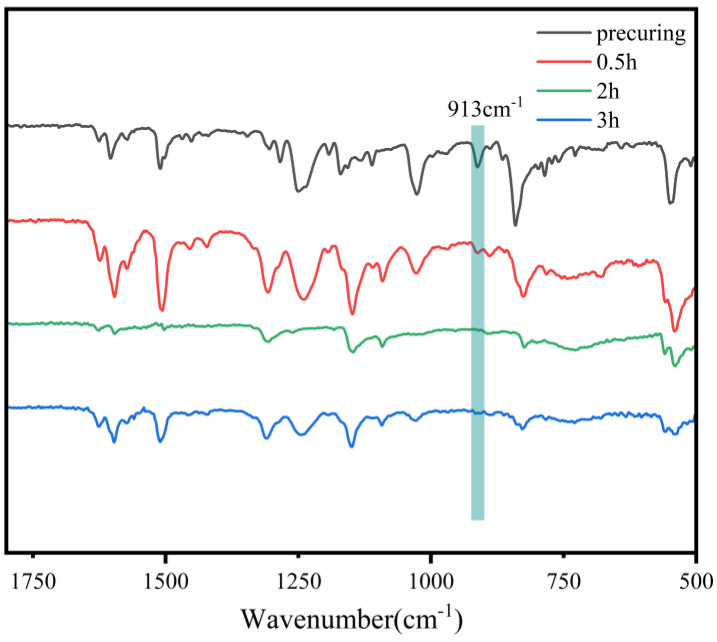
Infrared spectra of HBAP-EP/SAA cured at 120 °C for different time periods.

HBAP-EP was reacted with SAA at 120 °C for several hours. Figure 6 displays the infrared spectra collected at different curing durations at 120 °C. The characteristic peak of epoxy groups essentially vanished after 2 h of curing, thereby establishing the curing conditions for HBAP-EP. To test the effect of curing temperature on the crystallization properties of the cured material, HBAP-EP and SAA with the same stoichiometric amounts of active hydrogen and epoxy groups were weighed and heated for curing reaction in a forced-air oven at 120 °C, 140 °C, and 160 °C for 2 h.

**Figure 7:**
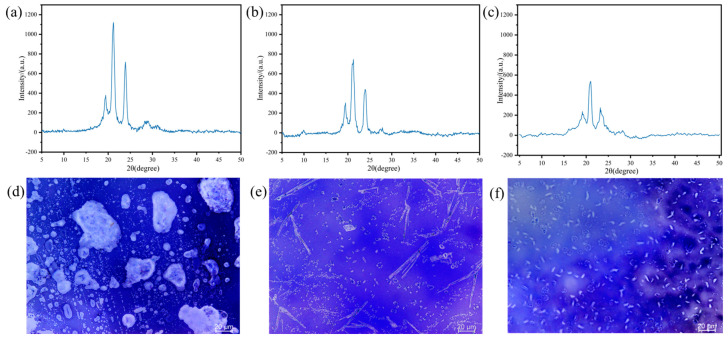
XRD curves of HBAP-EP/SAA cured resin at (**a**) 120 °C, (**b**) 140 °C, and (**c**) 160 °C, and the corresponding POM images directly underneath at (**d**) 120 °C, (**e**) 140 °C, and (**f**) 160 °C.

Figure 7 displays the XRD images and corresponding POM images of the monomer after curing at 120 °C, 140 °C, and 160 °C, respectively. The liquid crystal fractions, calculated based on the XRD data, are 14.4%, 8.24%, and 5.94% for the three samples, respectively. The crystallinity gradually decreases as the curing temperature increases. The striped texture visible in the field of view indicates the formation of nematic liquid crystals in HBAP-EP during the curing process. Consequently, HBAP-EP is an epoxy resin exhibiting liquid crystal behavior throughout the curing process.

In optical photographs, the system cured at 160 °C demonstrates a lower degree of mesomorphic domains. Resins cured at lower temperatures (120 °C, 140 °C) exhibit a higher liquid crystal fraction. During the curing process, monomers must assemble into an ordered structure via the self-assembly of mesogenic units. As the temperature rises, molecular motion intensifies, leading to a faster reaction rate. However, when the temperature exceeds an optimal range, the rate at which the resin forms liquid crystals may not keep pace with the curing rate, ultimately resulting in reduced crystallinity after curing [27]. As the curing temperature decreases, the size of liquid crystal domains significantly increases. Higher curing temperatures hinder the ordered arrangement of mesogenic units in liquid crystal epoxy resin (LCE).

### 3.5. Mechanical Properties of Cured Materials

Test the lap shear strength of aluminum alloy test samples, with a plate length of 100 mm and a lap area (length × width) of 12.5 mm × 25 mm. The shear strength is calculated according to Formula (3).

(3) $$\tau =\frac{P}{BL}$$

In the formula, τ represents the lap tensile shear strength of the adhesive, measured in Mpa. P denotes the maximum load at shear failure of the specimen, measured in N. B signifies the width of the specimen lap interface, measured in mm. L indicates the length of the specimen lap interface, measured in mm.

The fracture toughness of thermosetting plastics is measured at room temperature using the three-point bending method, in accordance with GB4161-1984. The dimensions of the sample are length × width × height = 48 mm × 12 mm × 6 mm. When the specimen width (w) is 12 mm, the pre-crack length ratio falls within the range of 0.50 to 0.75 w. The calculation formula for the fracture toughness J_IC_ of thermosetting plastics is as follows:

(4) $$J_{IC}=\frac{Af(a_{0}/w)}{Bb_{0}}$$

In this formula, J_IC_ represents the fracture toughness of thermosetting plastics, measured in kJ·m^−2^; A denotes the area under the load-opening displacement curve; B is the thickness of the specimen, in millimeters; a_0_ stands for the pre-crack length; W represents the width; b_0_ is obtained by subtracting a_0_ from W; and f(a_0_/W) is equal to 2.0.

As evident from Table 3, the fracture toughness of HBAP-EP/SAA resin surpasses that of regular bisphenol A epoxy resin, and this resin’s fracture toughness diminishes as the temperature rises. The lower curing temperature leads to the formation of more crystalline structures in the cured resin, and the enhancement of fracture toughness corresponds to the increase in liquid crystal (LC) content.

**Figure 8:**
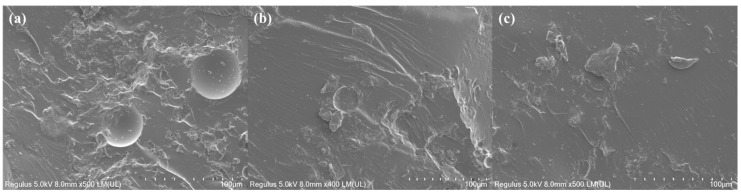
SEM images of HBAP-EP resin cured with SAA at different temperatures: (**a**) 120 °C, (**b**) 140 °C, (**c**) 160 °C.

The fracture surfaces of the resins obtained at different curing temperatures were observed using scanning electron microscopy (SEM), as shown in Figure 8. As the curing temperature decreases, the SEM images reveal a significant increase in the roughness of the fracture surface. This increase corresponds to an increase in the LC fraction and aligns well with the trend of changes in fracture toughness.

At a curing temperature of 120 °C, HBAP-EP/SAA displays nematic liquid crystal domains of larger size and higher orientation degree (Figure 7d). These ordered regions act as physical cross-linking points, effectively inducing craze and deflection crack paths, thereby absorbing more fracture energy. In addition, the π-π stacking and chemical bonding interactions between the liquid crystal (LC) and non-LC regions further hinder crack propagation, leading to increased roughness of the fracture surface (Figure 8a). However, using higher curing temperatures leads to a reduction in the size of the liquid crystal region and a decrease in orderliness, resulting in stress dispersion, a reduced craze induction rate, and decreased fracture toughness (Figure 8b,c). Due to its amorphous and isotropic nature, ordinary epoxy resin lacks a crystalline structure, and its molecular movement is restricted. This manifests as inherent brittleness, leading to poor mechanical properties [31].

**Figure 9:**
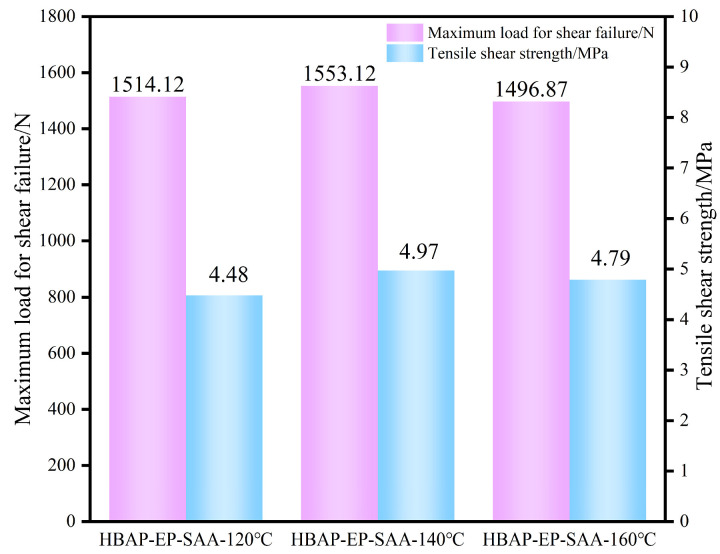
Bar chart of tensile shear strength of HBAP-EP-SAA resin.

Generally, epoxy resin structures contain hydroxyl and ether bonds, which endow it with high adhesion. These polar groups can generate electromagnetic forces at adjacent interfaces. During the curing process, with the chemical reaction of the curing agent, hydroxyl and ether bonds can be further formed. Therefore, epoxy adhesives not only have high cohesion but also strong adhesion, exhibiting strong bonding strength to many materials such as metals, plastics, glass, wood, fibers, etc. They are commonly known as “universal adhesives”. As shown in Figure 9, the mean lap tensile shear strength of Al alloy plates cured with SAA-HBAP-EP resin can reach 4.87 MPa, indicating that this liquid crystal polymer can serve as an adhesive for metals, effectively adhering to metal substrates.

### 3.6. Thermal Conductivity

The sample was tested according to the GB/T 32064-2015 standard, with a probe radius of 6.4 mm, a test duration of 160 s, and an output power of 0.25 W. Prepare two flawless, smooth round slices of the sample, each with a diameter of 3 cm and a height of 2 mm. Ensure that both slices are free from any obvious defects. Once the surfaces of the two samples are in close contact with the probe, proceed with the measurement. The initial state should be restored for each test, with an interval of no less than 5 min between tests. The test should be repeated three times, and the average value of the test results should be taken. The thermal conductivity should be calculated according to Formula (5).

(5) $$\lambda =\alpha C_{v}$$

In the formula, λ represents the thermal conductivity, with in units of W·m^−1^·K^−1^; α denotes the thermal diffusion coefficient in units of mm^2^·s^−1^; and C_υ_ signifies the specific heat capacity per unit volume of the sample in units of MJ·m^−3^·K^−1^.

A higher LC fraction in the 120 °C system indicates a more ordered molecular arrangement, leading to reduced phonon scattering and an extended mean-free path. This, in turn, creates a more continuous heat conduction pathway, thereby enhancing thermal conductivity. On the contrary, an increased curing temperature reduces the liquid crystal size and disrupts the thermal conduction path, thereby decreasing the thermal conductivity.

As can be seen from Table 4, as the curing temperature decreases, the LC fraction of the resin increases, and so does the thermal conductivity of the resin. The thermal conductivity increases from 0.2674 W·(m·K)^−1^ in the 160 °C system to 0.3229 W·(m·K)^−1^ in the 120 °C system, which is 1.54 times that of ordinary bisphenol A epoxy resin (0.19–0.2 W·(m·K)^−1^) [27]. In the study conducted by Guo et al., they synthesized liquid crystalline epoxy resins with lateral substituents and cured them using aromatic amines or anhydrides. The thermal conductivity of the cured samples was found to be 0.29 W·(m·K)^−1^ [28]. The thermal conductivity of the samples in this study surpasses that of their counterparts. While Kim et al. did synthesize an epoxy resin containing mesogenic units of methyleneimine, which exhibits high thermal conductivity, their synthesis method is intricate and the curing conditions are demanding: it necessitates hot pressing under high-temperature and high-pressure conditions, making it less conducive to process control in production [22,23].

Although the LC phase was also formed in the 160 °C system, the LC fraction was only 5.94%, and the thermal conductivity was relatively low. The ordered structure is solidified and fixed, forming a liquid crystal domain structure and enhancing the thermal conductivity of the resin itself. As the LCE content increases, the degree of liquid crystal order also increases, resulting in an increase in thermal conductivity.

Although curing at 120 °C yields a higher LC fraction and improved performance, it also results in a narrowed processing window. HBAP-EP exhibits a liquid crystal phase only within the temperature range of 93.5–118.9 °C. When the temperature falls below 120 °C, the reaction rate slows down, and the fluidity of the epoxy monomer deteriorates, resulting in incomplete curing. If the temperature exceeds a certain threshold, it can disrupt the liquid crystal structure. Hence, 120 °C is the optimal curing temperature for attaining a high LC fraction and ensuring good processing performance.

## 4. Conclusions

In this paper, a bisphenol compound, HBAP-EP, featuring C=N double bonds as bridging groups connecting two benzene rings as mesogenic units, was successfully synthesized. The successful synthesis of the monomer was confirmed by infrared spectroscopy, nuclear magnetic resonance, and X-ray diffraction. HBAP-EP with high crystallinity and low epoxy-equivalent weight was obtained. The synthesis process was safe, the post-treatment was simple, and the yield reached 91.24%. The crystallinity was 84.62%, and the epoxy-equivalent weight was 163.95 g·eq^−1^. After curing at 120 °C for 2 h, the liquid crystal fraction of the obtained resin reached 14.4%, and this fraction decreased as the curing temperature increased. The fracture toughness of the resin obtained through curing at 120 °C, 140 °C, and 160 °C is higher than that of ordinary bisphenol A epoxy resin, and it decreases as the curing temperature increases. The average lap shear strength between resin and Al alloy sheets can reach 4.87 MPa. As the curing temperature decreases, the LC fraction of the resin increases, and so does its thermal conductivity. The thermal conductivity of HBAP-EP/SAA resin cured at 120 °C can reach 0.3229 W·(m·K)^−1^, which is 1.54 times that of ordinary bisphenol A epoxy resin.

## Figures and Tables

**Table 1:** Concentration of sodium hydroxide–ethanol standard solution.

M (g)	V_1_ (mL)	V_2_ (mL)	M (g·mol^−1^)	C_(NaOH)_ (g·mol^−1^)
0.7511	1.15	31.23	204.22	0.1237

**Table 2:** Calculation of HBAP-EP epoxy equivalent (EEW).

Sample Mass (g)	V_0_ (mL)	V (mL)	EEW (g·eq^−1^)
0.3257	39.88	23.84	163.88
0.3261	39.69	23.62	164.01
0.3254	39.80	23.78	163.97

**Table 3:** Determination of fracture toughness of HBAP-EP/SAA resin by three-point bending method.

Resin	A (0.0098 J)	B (mm)	B_0_ (mm)	J_IC_ (KJ·m^−2^)	LC Score (%)
DGEBA/SAA-120 °C	0.49	5.63	4.27	0.39	0
HBAP-EP/SAA-120 °C	1.22	5.23	4.89	0.93	14.4
HBAP-EP/SAA-140 °C	0.81	5.54	4.32	0.66	8.24
HBAP-EP/SAA-160 °C	0.75	5.25	5.01	0.55	5.94

The LC score is calculated from XRD data using data processing software.

**Table 4:** Thermal conductivity of HBAP-EP resin cured with SAA at different temperatures.

Resin	C_υ_ (MJ·m^−3^·K^−1^)	α (mm^2^·s^−1^)	λ (W·m^−1^·K^−1^)
HBAP-EP/SAA-120 °C	2.2043	0.1465	0.3229
HBAP-EP/SAA-140 °C	2.7376	0.1168	0.3197
HBAP-EP/SAA-160 °C	2.5298	0.1057	0.2674

## Data Availability

The original contributions presented in this study are included in the article. Further inquiries can be directed to the corresponding authors.
